# High temperature and vapor pressure deficit aggravate architectural effects but ameliorate non-architectural effects of salinity on dry mass production of tomato

**DOI:** 10.3389/fpls.2015.00887

**Published:** 2015-10-20

**Authors:** Tsu-Wei Chen, Thi M. N. Nguyen, Katrin Kahlen, Hartmut Stützel

**Affiliations:** ^1^Department of Vegetable Systems Modelling, Institute of Horticultural Production Systems, Leibniz Universität HannoverHannover, Germany; ^2^Department of Vegetable Crops, Hochschule Geisenheim UniversityGeisenheimw, Germany

**Keywords:** dynamic functional-structural plant model, canopy architecture, canopy photosynthesis, allometric relationship, tomato, high temperature, salinity, stress combination

## Abstract

Tomato (*Solanum lycopersicum* L.) is an important vegetable crop and often cultivated in regions exposed to salinity and high temperatures (HT) which change plant architecture, decrease canopy light interception and disturb physiological functions. However, the long-term effects of salinity and HT combination (S+HT) on plant growth are still unclear. A dynamic functional-structural plant model (FSPM) of tomato was parameterized and evaluated for different levels of S+HT combinations. The evaluated model was used to quantify the contributions of morphological changes (architectural effects) and physiological disturbances (non-architectural effects) on the reduction of shoot dry mass under S+HT. The model predicted architectural variables with high accuracy (>85%), which ensured the reliability of the model analyses. HT enhanced architectural effects but reduced non-architectural effects of salinity on dry mass production. The stronger architectural effects of salinity under HT could not be counterbalanced by the smaller non-architectural effects. Therefore, long-term influences of HT on shoot dry mass under salinity were negative at the whole plant level. Our model analysis highlights the importance of plant architecture at canopy level in studying the plant responses to the environments and shows the merits of dynamic FSPMs as heuristic tools.

## Introduction

Salinity is a severe problem for agricultural production in many parts of the world (Munns and Tester, 2008). Salinity stress has negative effects on plant morphology, which may reduce light interception of the canopy (referred to as architectural effects), and physiology, which is a combination of osmotic stress and ionic stress (Rajendran et al., 2009; Harris et al., 2010; referred to as non-architectural effects). Osmotic stress affects plant growth and development due to low water potential in the root zone (Munns, 1993; Hasegawa and Bressan, 2000; Munns, 2002). The primary architectural effects of osmotic stress are the decreases in leaf size and internode length which reduce light interception (Al-Karaki, 2000; Najla et al., 2009; Rajendran et al., 2009). The non-architectural effects of osmotic stress are the reduction of stomatal and mesophyll conductance that restrict CO_2_ diffusion into the chloroplast and reduce photosynthesis rate per unit leaf area (James et al., 2002; Maggio et al., 2007; Pérez-López et al., 2012). Therefore, whole plant photosynthesis and dry mass production may be restricted by architectural and non-architectural effects of osmotic stress, at the canopy and leaf levels. Ionic stress results from the accumulation of ions in leaf cells above certain concentrations. High ion concentrations in the leaf cells are toxic, disturb stomatal regulation, and reduce photosynthetic capacity (James et al., 2002; Rajendran et al., 2009). Therefore, ionic stress enhances the non-architectural effect of salinity.

Tomato is one of the most widely produced and consumed vegetable crops and is classified as moderately sensitive to salinity (Cuartero and Fernández-Muñoz, 1998). In tomato, the most obvious and visible symptoms of salinity are the changes in plant architectural traits, e.g., leaf area (Li and Stanghellini, 2001; Maggio et al., 2004), internode length (Romero-Aranda et al., 2001; Najla et al., 2009; Zribi et al., 2009), and leaf angle (Jones and El-Beltagy, 1989; Shibli et al., 2007). By using a dynamic functional-structural plant model (FSPM, Vos et al., 2010; DeJong et al., 2011), where the detailed 3D architecture of plant and physiological functions were combined, Chen et al. (2014) have demonstrated that changes in individual architectural traits may affect dry mass production by up to 20% and that the sensitivity of dry mass production to architectural modifications is not only trait but also temperature dependent. This study highlights that the architectural effects on dry mass production are stronger than suggested in the literature (e.g., 8% in Sarlikioti et al., 2011 and 5% in Song et al., 2013). Their results raise the question to which extend the reductions of dry mass production under salinity result from architectural effects and light interception? However, it is experimentally impossible to assess the pure architectural effects of salinity on dry mass production because in reality they occur together with non-architectural effects such as the reduction of stomatal and mesophyll conductance.

The degree to which architectural traits are influenced by salinity is genotype-dependent. For example, in comparison with non-stressed plants, reductions of leaf number and leaf area have been shown to range between 0–9% and 7.4–17.1%, per 10 mM NaCl in the solution (Supplementary Table S1). Jones and El-Beltagy (1989) reported a threefold difference in the change of leaf angle due to salinity between tomato genotypes. These experimental results suggest that there should be a wide spectrum of salt-induced morphological changes in the tomato genome. Although these changes have received some attentions, no study, to our knowledge, has quantified the effects of these alterations on light interception and, as a result, on dry mass production.

Salinity is often associated with high temperatures (HT; Rivero et al., 2014; Suzuki et al., 2014). It is surprising that the combined effects of salinity and HT are rarely studied (Colmenero-Flores and Rosales, 2014). HT is often associated with high vapor pressure deficit (VPD), which reduces the leaf elongation rate (Reymond et al., 2003). Therefore, it is not surprising that Keleks and Oncel (2002) showed that HT aggravates the salinity effects on leaf and root length of wheat seedlings (architectural effects). However, their results contradict with a recent study in tomato (Rivero et al., 2014) addressing the short-term responses of tomato to the combined effects of salinity and HT (first 72 h after exposing to 120 mM NaCl in nutrient solution of a hydroponic system). The findings of Rivero’s study suggest that heat stress ameliorates the negative non-architectural effects of salinity (less ion uptake and higher photosynthesis rate per leaf area) and highlight the fact that the combined impact of two stresses must not be the sum of their individual effects. It seems as if the results of Rivero et al. (2014) are not consistent with the previous findings of Keleks and Oncel (2002). However, this can be explained by the different target traits in their studies: Keleks and Oncel (2002) investigated the combined effects on architectural traits (leaf and root length) and Rivero et al. (2014) focused on non-architectural traits (stomatal conductance, photosynthesis, and Na^+^ uptake). Combining the knowledge from these studies, it seems that HT aggravates the architectural effects of salinity but ameliorates the non-architectural effects. However, the magnitudes of these aggravation and amelioration and the long-term effects (more than weeks) of them on dry mass production at the whole plant level are still unknown. The aim of this paper is to test the hypothesis that HT increases architectural effects of salinity but reduces non-architectural effects of salinity by combining data from five experiments and analyses from a dynamic FSPM. Furthermore, the sensitivity of dry mass production to architectural parameters in the FSPM was analyzed to investigate the importance of architectural traits on stress tolerance.

## Materials and Methods

### Model Structure

Details of the dynamic FSPM describing the structure and growth of a tomato crop can be found in the Figure 1 in Chen et al. (2014) and in the Supplementary Materials. In short, the whole plant architecture was reconstructed by a parametric L-System using the *lpfg* plant modeling program (Prusinkiewicz and Lindenmayer, 1990; Karwowski and Lane, 2008). Each leaf consisted of seven leaflets with a phyllotaxis angle of 144° (Najla et al., 2009). Each leaflet was represented by a rhombus. The virtual tomato canopy comprised 16 plants (4 × 4) with row and plant distances equal to one meter, representing the setup of the experiment for model evaluation (see below). In each step of simulation (1 day), growth and elongation of the leaves and internodes in the canopy were calculated based on the environmental data [salinity level, greenhouse temperature, VPD, and photosynthetically active radiation (PAR) above the canopy] and then the canopy architectures were sent to the light simulation model *QuasiMC* (Cieslak et al., 2008) to estimate light absorption of each leaflet in the canopy. This light model is based on Quasi-Monte Carlo algorithm, a path trace algorithm, and the light source was an approximation of the sky (see Figure 2 in Kahlen and Stützel, 2011), calibrated by the location (Hannover, Germany, lat.52°23′ N, long. 9°37′ E) and day of year of the experiment (Cieslak et al., 2008). The light source emitted more than 1 million rays and each ray had a recursion depth of 10 reflections. Further information about the goodness of the light model and the communications between light and architecture model can be found in Cieslak et al. (2008) and Kahlen and Stützel (2011). The adaxial and abaxial sides of tomato leaves reflect 7.3 and 12.7% of incident light and transmit 2.4 and 2.5% of incident light, respectively (Chen et al., 2014). The absorbed light of each leaf was used to calculate the dry mass production. The virtual ground was a rectangle, reflecting 80% of incident light without transmittance. The canopy and ground setups were identical to the setups of the experiment for model evaluation and model analyses (see below).

**FIGURE 1:**
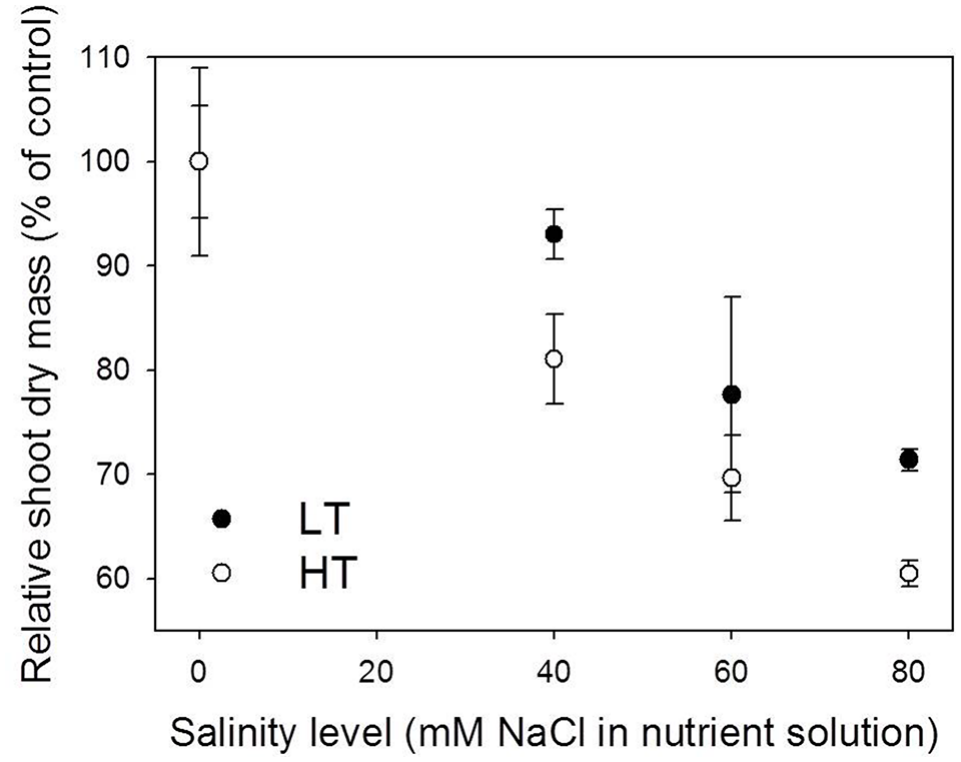
**Effect of salinity on shoot dry mass on day 77 after the first leaf appearance under 22/18°C (LT) and 32/28°C (HT) day/night temperature conditions.** The absolute shoot dry mass of control plants (0 mM NaCl) under LT and HT conditions was 674.5 and 534.8 g, respectively (see the points with the highest shoot dry mass in **Figure 4A**). Data were obtained from the experiment for model evaluation (*n* = 4).

**FIGURE 2:**
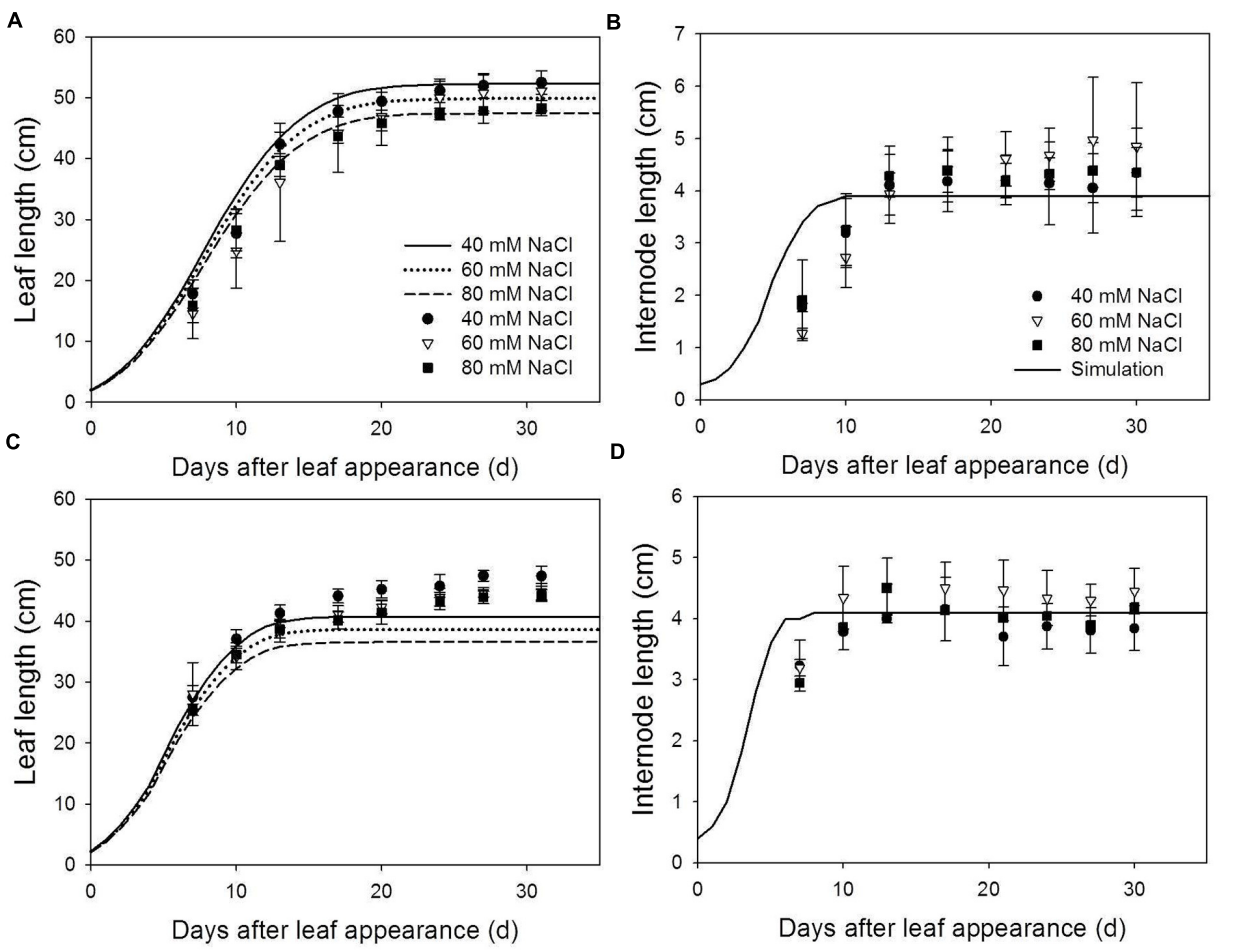
**Measured (symbols) and simulated (lines) leaf length **(A,B)** and internode length **(C,D)** at 22/18°C (LT, **A,C**) and 32/28°C (HT, **B,D**) day/night temperature regimes under 40 (circles), 60 (triangles) and 80 (squares) mM NaCl.** Since salinity has no effect on internode length, simulations for internode length under different salinity level were the same. Measured data were obtained from the 8th rank of the plants grown in the experiment for model evaluation (*n* = 4).

### Plant Materials for Model Parameterization

The dynamic FSPM of Chen et al. (2014) was for temperature effects on tomato plants (*Solanum lycopersicum* L. ‘Pannovy,’ Syngenta) grown under non-saline conditions. To implement the effects of salinity on plant architecture into the model, the same tomato cultivar was used in four experiments conducted in the growth chambers (Experiments 1–3) and greenhouses (Experiment 4) of Leibniz Universität Hannover, Germany. Experimental details can be found in the Supplementary Table S2.

The growth chamber experiments were designed to investigate the environmental effects (temperature, air VPD, and light intensity) on leaf and internode responses to salinity (Eqs S2, S6, S9, and S11). In these experiments, leaf length (cm) and internode length (cm) were measured by a ruler daily. The growth chamber experiments were set up with four levels of salinity (0, 20, 40, and 60 mM NaCl in nutrient solution for Experiment 1 and 0, 40, 60, and 80 mM NaCl in nutrient solution for Experiments 2 and 3) in combination with a variation of temperature, VPD and PAR. In the growth chambers, the experiments were arranged in split plot designs with environmental conditions as main plot factors and salt stress as the sub plot factor.

Experiment 4 was conducted in 2009 and was used to examine the effects of salinity on leaf shape, leaf angle, and curvature (Eqs S7, S8, S11), which were derived from weekly plant digitizing using a Fastrak 3D digitizer (Polhemus, Inc., Colchester, VT, USA). In Experiment 4, salinity treatments (for 4 weeks) were identical with those in Experiments 2 and 3 and air temperature, VPD and PAR in the greenhouses were recorded hourly. The greenhouse experiment was set up as a randomized complete block design with four replications and four plants per plot. Plant protection was applied when necessary.

### Plant Materials for Model Evaluation

To evaluate the model for tomato architecture under salinity stress, Experiment 5 was established in spring of 2010 in two greenhouses with 22/18 and 32/28°C day/night temperature, representing low temperature (LT) and HT conditions, respectively. Salinity treatments in Experiment 5 were identical with those in Experiments 2–4 and were applied on 21 day after the first true leaf appearance (DAFLA) for 7 weeks. In this experiment, leaf and internode length, leaf number, and plant height were recorded twice a week and the whole plant architectures were recorded by a Fastrak 3D digitizer weekly as in Experiment 4. Four plants per treatment were harvested on 28, 35, 43, 50, 56, 63, 70, and 77 DAFLA. Leaf area of the harvested plants were measured using a leaf area meter (LI-3100, LI-COR, Inc., USA) and then dried at 70°C for at least 96 h to determine dry mass. Further details of the experimental set-ups, cultivation schedule and weather data of the greenhouse experiments can be found in Supplementary Table S2 and in Chen et al. (2014).

### Simulations and Model Evaluation

Simulations started from the DAFLA and were run for 80 days under two different temperature regimes with the measured climate data from Experiment 5 and under four salinity levels (0, 40, 60, and 80 mM NaCl in the nutrient solution). Simulations were run five times with a randomized variation in phyllotaxis angle (144 ± 10°). At the organ level, measured and simulated leaf and internode growth over time was compared for rank 8. At the canopy level, measured and simulated leaf number, plant height (sum of all internode lengths of a plant), whole plant leaf area and shoot dry mass (above-ground dry mass, including leaves, stems, petioles, and fruits) were compared. Simulated and measured data were compared using root mean square deviation (RMSD), bias and accuracy (Kobayashi and Salam, 2000; Kahlen and Stützel, 2011).

### Estimating Relative Canopy Light Use Efficiency under Salinity Stress

Dry mass production by a leaf at time *t*, Δ*W*_l_(*t*) (g d^-1^), was the product of leaf area (*A*_l_(*t*), m^2^), light absorption of the leaf [*I*_abs_(*t*), J m^-2^ d^-1^, see Model Structure] and light use efficiency, 𝜀 (g CO_2_ J^-1^):

$$\Delta W_{1}(t)=\left(I_{abs}\right)(t).\epsilon (I_{abs}(t)).K_{T,x.}A_{1}\left(t\right)$$

where 𝜀(*I*_abs_(*t*)) is an empirical light-dependent function for tomato derived from Warren-Wilson et al. (1992) and is defined as the reference canopy light use efficiency, *k*_T,x_ is the effect of temperature and salinity on light use efficiency (the subscripts _T_ and _x_ indicate the temperature regimes and salinity levels, respectively). Daily increase in plant dry weight (Δ*W*_p_, g) was the integration of Δ*W*_l_ produced by all leaves and the plant dry weight (*W*_p_(*t*), g) was then the integration of Δ*W*_p_ with time. The shoot weight (*W*_sh_(*t*), g), was considered a constant proportion of *W*_p_(*t*) [*W*_sh_(*t*) = μ*⋅W*_p_(*t*), where μ is a partitioning factor of dry weight to above-ground organs]. To predict canopy dry mass production under various environmental conditions, using canopy light use efficiency has been demonstrated to be a robust approach (Kahlen and Stützel, 2011; Chen et al., 2014). Furthermore, it has been experimentally shown that estimated canopy light use efficiency reflects the environmental effects on it (Warren-Wilson et al., 1992; Hui et al., 2001; Benincasa et al., 2006). In Eq. 1, *k*_T,x_ represents the relative canopy light use efficiency, the integrated effects of the complicated interactions between temperature, osmotic and ionic effects on photosynthetic parameters (which are also related to ion transport to and ion accumulation in leaf). Here, we present a new method to estimate the changes in *k*_T,x_ during the growing period using the dynamic FSPM and measured allometric relationships between shoot dry mass and total leaf area.

Allometric relationships between plant traits have been shown from cell to population levels (Enquist et al., 1998; Harmens et al., 2000; Kahlen and Stützel, 2007; Niklas et al., 2009; John et al., 2013). For example, strong relationships between total leaf area and shoot dry mass have been widely reported (Bartelink, 1997; Gunn et al., 1999; Harmens et al., 2000; Niklas et al., 2009). Allometric relationships between measured total leaf area (*A*_n_) and shoot dry mass under non-stress conditions, *W*_sh,n_, were described by:

$$In\left(A_{n}\right)=P_{n}.In\left(W_{sh,n}\right)+q_{n}$$

where *p*_n_ and *q*_n_ are empirical coefficients for non-stress conditions. Since salinity slightly changes the allometric relationship between total leaf area and shoot dry mass (Poorter et al., 2012), coefficients *p_s_* and *q_s_* are estimated from leaf area and shoot dry weight under salinity (*A*_s_ and *W*_sh,s_, respectively):

$$In\left(A_{s}\right)=P_{s}.In\left(W_{sh,s}\right)+q_{s}$$

Data from LT and HT conditions were analyzed separately, because leaf and stem mass fractions of the whole plant mass, which have a strong influence on the slope (*p*) and intercept (*q*) parameters, are influenced by temperature (Poorter et al., 2012). Data collected from different salt levels were pooled because salinity is the environmental factor which has least effect on this allometry (Poorter et al., 2012), but analyzed separately for LT and HT.

A crop model where *W*_sh_ and total leaf area are accurately simulated should reflect the measured allometric relationships. Achieving accurate predictions of allometric relationships requires accurate predictions of (1) leaf growth dynamics, (2) leaf distribution in the space, and (3) canopy light use efficiency. Our model predicts leaf growth dynamics and leaf distribution with high accuracies (see Model Evaluation in the Results section and Chen et al., 2014) but uses a very simple function as the reference canopy light use efficiency [𝜀(*I*_abs_(*t*)) in Eq. 1], which can be influenced by leaf age, temperature (Gent and Seginer, 2012) and both, osmotic and ionic stress of salinity (James et al., 2002, 2008). Therefore, the ratio between measured dry mass production and simulated dry mass production using reference canopy light use efficiency represents the relative canopy light use efficiency, *k*_T,x_ (the subscript _T_ and _x_ denote temperature or salinity conditions, respectively):

$$k_{T,x}\left(t\right)=(W_{sh,m}(t+1)-W_{sh,m}\left(t\right))/(W_{sh,r}(t+1)-W_{sh,r}\left(t\right))$$

where *W*_sh,m_ and *W*_sh,r_ are the shoot dry mass at time *t* based on the measured allometric relationships (Eqs 2a,b) and simulations with reference canopy light use efficiency, respectively. The steps for time *t* were 28, 35, 43, 50, 56, 63, 70, and 77 DAFLA. By running the model for unstressed conditions with *k*_T,0_⋅𝜀(*I*_abs_(*t*)) instead of 𝜀(*I*_abs_(*t*)), the simulated allometric relationships between total leaf area and shoot dry mass should fit the measured relationships (Eq. 2a). The same, by running the model for stress conditions with *k*_T,x_⋅𝜀(*I*_abs_(*t*)), the simulated allometric relationships should match Eq. 2b. Here we want to emphasize that the biological meaning of *k*_T,x_ is the relative photosynthetic capacity of a whole plant, an outcome of combined effects of temperature, salinity, leaf and canopy age. For this reason, temperature and salinity effects on relative canopy light use efficiency were further dissected:

$$k_{T,x}=k_{KT,0}.k_{HT}.k_{x}$$

where *k*_LT,0_ is the relative canopy light use efficiency under LT and non-salinity condition, *k*_HT_ is the effects of HT (set to 1 for LT conditions) and *k*_x_ is the effects of *x* mM NaCl in the nutrient solution (set to 1 for 0 mM NaCl).

### Dissecting the Architectural and Non-architectural Effects of Salinity

The architectural and non-architectural effects of salinity on dry mass production (*R*_a,x_ and *R*_n,x_, respectively, %) at *x* mM NaCl was calculated by:

$$R_{a,x}=(W_{sh,0}-W_{sh,a})/W_{sh,0}$$

$$R_{n,x}=(W_{sh,a}-W_{sh,x})/W_{sh,0}$$

where *W*_sh,0_ is the shoot dry mass simulated with the control architecture and the control light use efficiency, *k*_T,0_⋅𝜀(*I*_abs_); *W*_sh,a_ is the shoot dry mass simulated with the architecture being affected by *x* mM NaCl but with the control light use efficiency; and *W*_sh,x_ is the shoot dry mass simulated with architecture and light use efficiency affected by *x* mM NaCl. The term *W*_sh,0_ – *W*_sh,a_ in Eq. 4a represents the difference in shoot dry mass resulted from salinity effects on total leaf area, leaf angle, and canopy light interception, the architectural effects. The term *W*_sh,a_ – *W*_sh,x_ in Eq. 4b is the reduction of shoot dry mass due to the salinity effects on light use efficiency, the non-architectural effect.

### Sensitivity of Shoot Dry Mass to Architectural Traits under Salinity

The FSPM was used to test the sensitivity of dry mass production to architectural parameters under salinity. The model with light use efficiency equal to 𝜀_x_ was used for quantifying the effects of architectural traits on light interception and dry mass production under 40 and 80 mM NaCl, separately for both temperature regimes. Leaf number, leaf area, internode length and leaf angle were chosen for the analyses because they are most frequently reported to be influenced by salinity. The testing range for each trait was determined according to the values reported in the literature (Supplementary Table S1): the reduction of leaf number was by 3, 6, and 9% per 10 mM NaCl in the solution. Reduction of internode length was by 2, 4, and 6% per 10 mM NaCl in the solution. To evaluate this effect of salinity on leaf area, sensitivity of leaf elongation rate to salinity (parameter *c*_El,max_ in the Eqs S2a and S2b) was simulated with 50–150% of the reference value. Leaf angle was simulated with 70–130% of the reference values (100%). Only one morphological trait was changed for each analysis.

## Results

### High Temperatures and Vapor Pressure Deficit Aggravate Salinity Effects on Shoot Dry Mass

It is important to stress that the temperature effects presented in this study were the combined effects of temperature and VPD since they were highly correlated in the experiments (*R*^2^ = 0.71, data not shown) and both of them influenced the leaf elongation (Eq. S2). In Experiment 5, reduction in shoot dry mass due to salinity stress under HT was stronger than under LT on day 77 DAFLA. In comparison with the tomato plants grown under control conditions, measured shoot dry mass of plants grown under 40, 60, and 80 mM NaCl was reduced by 6.1, 22.5, and 28.6%, respectively, under LT, and 19.9, 30.3, and 39.4%, under HT conditions (**Figure 1**). On day 77 DAFLA, total leaf number and plant height were not different between salinity treatments (data not shown).

### Model Evaluation

The model described the reduction of leaf length due to salinity under LT very well (**Figure 2A**). At HT, final leaf length was underestimated by 2.7–4.8 cm (**Figure 2B**). Predicted leaf lengths had accuracies higher than 85% (**Table 1**). Salinity had no effect on internode length for both LT and HT (**Figures 2C,D**) and the model overestimated the leaf length under LT and internode growth in the early phase. This resulted in lower accuracies in predicting internode length (**Table 1**). However, standard deviations of the measured final internode lengths were high and the difference between measured and simulated final internode lengths were less than 1 cm (**Figures 2C,D**).

**Table 1:** Statistical analysis for the comparison between simulated and measured data for organ level and canopy level for the whole duration of leaf and plant growth at 22/18°C (LT) and 32/28°C (HT) day/night temperature conditions (*L*_l_, leaf length of rank 8; *L*_i_, internode length of rank 8; *A*_s_ and *W*_sh,a_ are, respectively, total leaf area and shoot dry weight; RMSD, root mean square deviation).

Salinity level	Traits	LT			HT		
			
		RMSD	Bias	Accuracy (%)	RMSD	Bias	Accuracy (%)
40 mM NaCl	*L*_l_	2.87	-2.08	93.3	4.19	2.69	89.6
	*L*_i_	0.68	-0.09	81.9	0.44	-0.35	88.2
	*A*_s_	2124	1570	87.8	1048	932	91.3
	*W*_sh,a_	38.4	30.7	86.3	16.0	5.6	92.5
60 mM NaCl	*L*_l_	4.23	-2.83	89.4	4.14	3.42	89.6
	*L*_i_	1.08	0.10	72.6	0.42	0.02	90.2
	*A*_s_	1865	1488	88.1	455	-222	95.7
	*W*_sh,a_	23.1	2.5	90.8	29.8	-20.5	84.2
80 mM NaCl	*L*_l_	1.79	-0.98	95.5	5.31	4.75	86.5
	*L*_i_	0.68	-0.04	82.4	0.42	-0.13	89.4
	*A*_s_	1547	1391	89.0	684	343	93.1
	*W*_sh,a_	37.6	-20.8	83.0	36.5	-29.3	78.0


**FIGURE 3:**
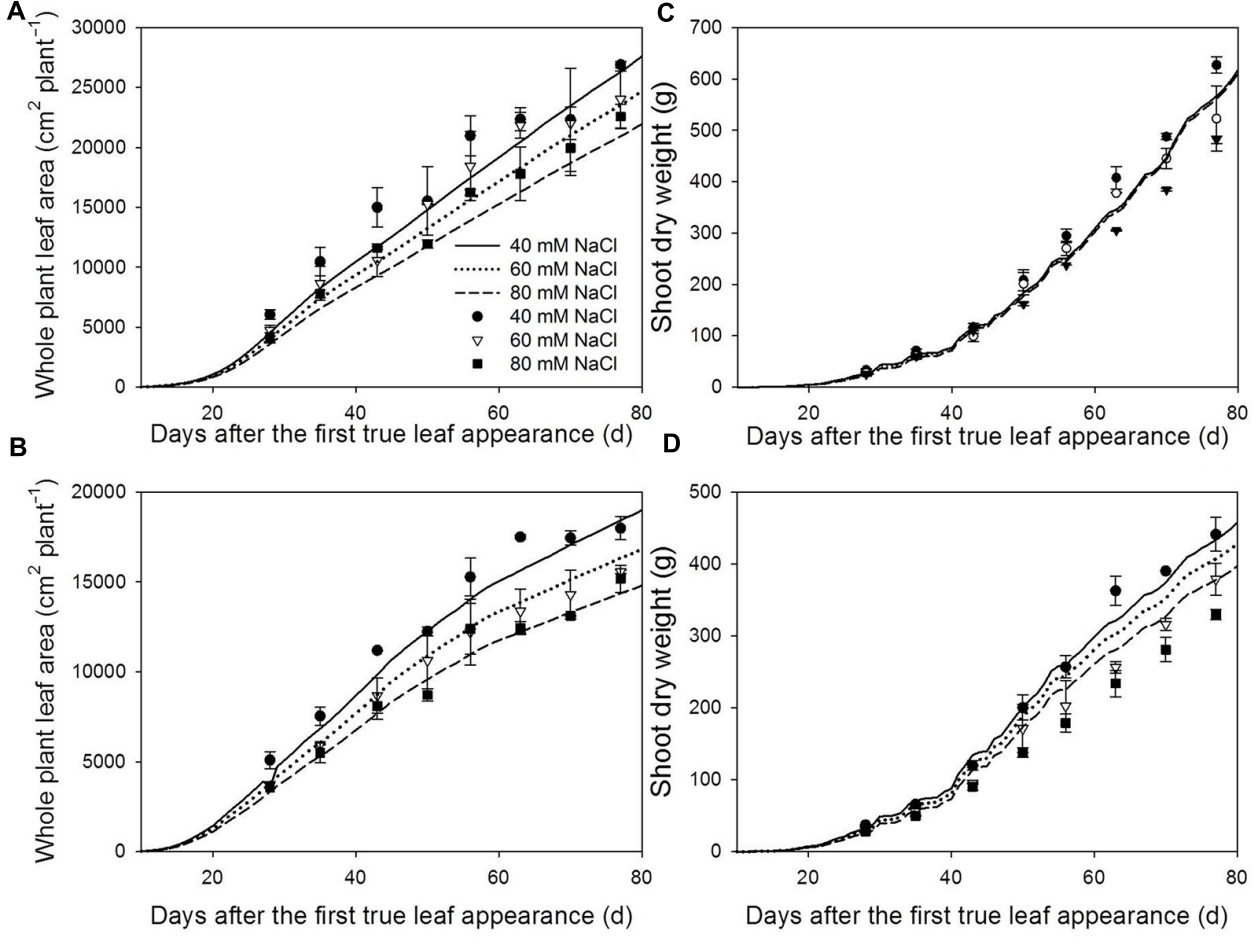
**Measured (symbols) and simulated (lines) total plant leaf area **(A,B)** and shoot dry weight **(C,D)** at 22/18°C (LT, **A,C**) and 32/28°C (HT, **B,D**) day/night temperature regimes under 40 (circles), 60 (triangles) and 80 (squares) mM NaCl.** The measured and simulated total leaf area and shoot dry weight of non-stress plants can be found in Chen et al. (2014). Measured data are from the plants grown in the experiment for model evaluation (*n* = 4). The simulated shoot dry weights were the results with the reference light use efficiency [𝜀(*I*_abs_(*t*)) in Eq. 1].

Both measured and simulated results show that salinity reduced total leaf area under LT (**Figure 3A**) and HT (**Figure 3B**). For all salinity levels and both temperature conditions, the simulated total leaf area was well in accordance with the measurements (accuracies > 87%, **Table 1**). The measured shoot dry mass under 80 mM NaCl was 23% less than under 40 mM NaCl at LT but, interestingly, the simulated 16% reduction of total leaf area under LT (**Figure 3A**) reduced the simulated dry mass production by only 1.1% (**Figure 3C**). In contrast, the simulated shoot dry mass under 80 mM NaCl was 13% less than under 40 mM NaCl at HT (**Figure 3D**). The accuracies of the simulated shoot dry mass with reference canopy light use efficiency (Eq. 1) decreased with the salinity level for both LT and HT conditions (**Table 1**). The random factor in the model only resulted in a very slight difference (<1%) between simulations. Therefore, the simulated data shown in **Figures 2** and **3** were the results of one simulation.

### Allometric Relationships between Shoot Dry Mass and Total Leaf Area

**FIGURE 4:**
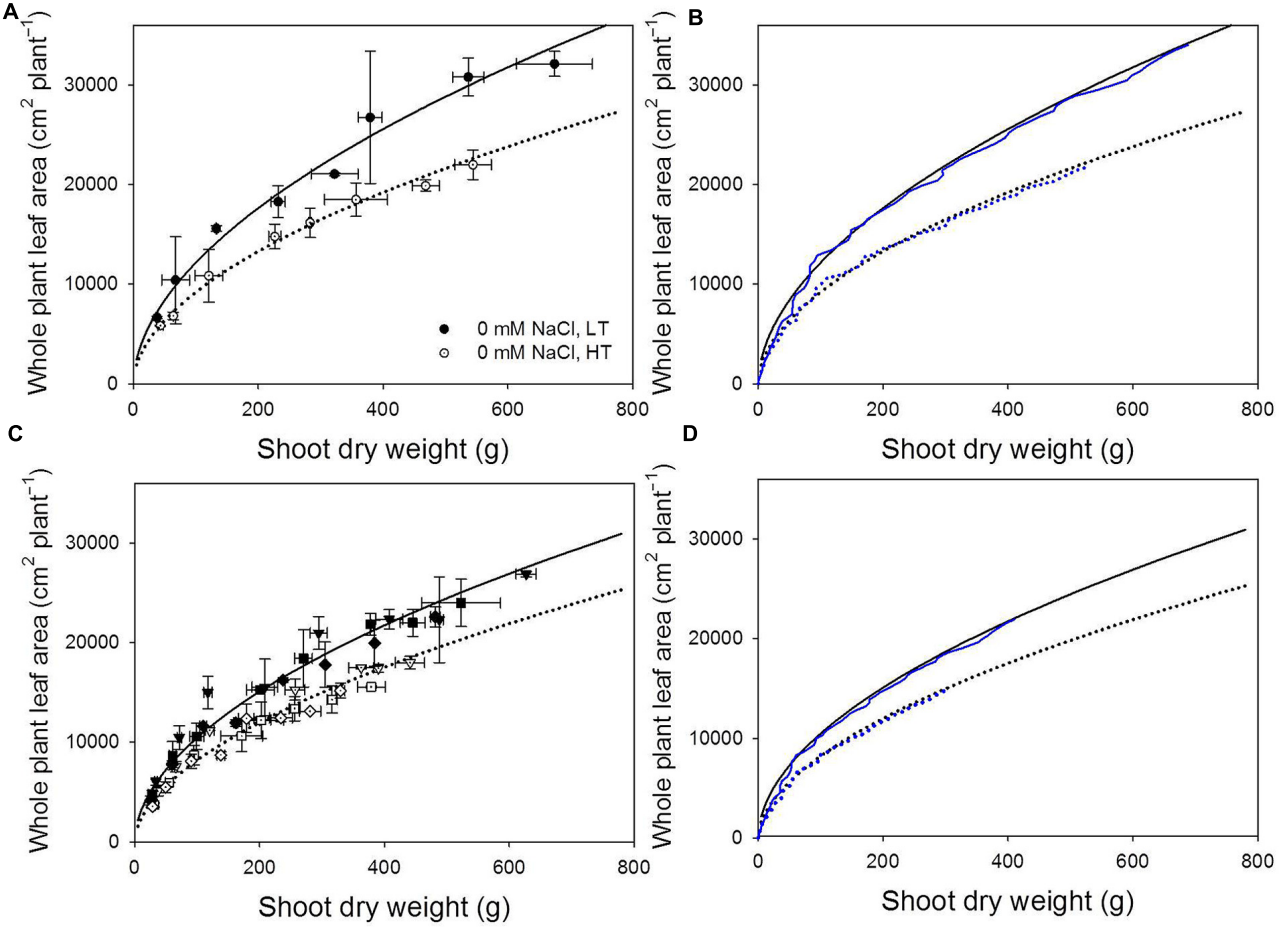
**Measured **(A,B)** and simulated **(C,D)** allometric relationships between shoot dry weight and whole plant leaf area (cm^2^ plant^-1^) at 22/18°C (LT, closed symbols and solid lines) and 32/28°C (HT, open symbols and dotted lines) day/night temperature regimes under non-stress **(A)** and under 40, 60, and 80 mM NaCl (reversed triangle, square, and rhombus symbols, respectively; **B**).** Black lines represent the regression lines fitted by the data collected from the experiment for model evaluation according to Eq. 2a (non-stress, **A,C**) and Eq. 2a (salinity stress, **B,D**). The blue lines show the simulated allometric relationships using light use efficiency equal to *k*_T,x_⋅𝜀(*I*_abs_(*t*)).

Significant allometric relationships between total shoot dry mass and total leaf area were found (**Figures 4A,B**, in all cases, *R*^2^ > 0.95, *p* < 0.0001). Running the model with canopy light use efficiency equal to *k*_T,x_⋅𝜀(*I*_abs_) instead of the reference light use efficiency, the simulated allometric relationships matched the measured relationships (**Figures 4C,D**).

### Temperature and Salinity Effects on the Relative Canopy Light Use Efficiency

Under LT conditions, the relative canopy light use efficiency (*k*_T,x_ in Eq. 3b) was higher than 1 for days 29–56 and decreased with time (**Table 2**). Furthermore, HT reduced canopy light use efficiency, *k*_HT_. Light use efficiency decreased with the increasing salinity level under both LT and HT condition (*k*_40_ < *k*_60_ < *k*_80_) and with time after exposure to salinity (**Table 2**). The degree of this decrease with time under 60 and 80 mM was stronger than under 40 mM NaCl.

**Table 2:** Relative canopy light use efficiency, *k*_T,x_ (Eq. 3a) and effects of high temperature (*k*_HT_, Eq. 3b) and *x* mM NaCl salinity (*k*_x_, Eq. 3b) on canopy light use efficiency under 22/18°C (LT) and 32/28°C (HT) day/night temperature conditions.

	0 mM		40 mM		60 mM		80 mM	
				
DAFLA	LT	HT	LT	HT	LT	HT	LT	HT
	
	*k*_LT,0_	*k*_HT,0_	*k*_LT,40_	*k*_HT,40_	*k*_LT,60_	*k*_HT,60_	*k*_LT,80_	*k*_HT,80_
29–35	1.38	1.33	1.61	1.42	1.32	1.24	1.10	1.08
36–43	1.60	1.59	1.76	1.69	1.41	1.45	1.10	1.24
44–50	1.02	0.91	1.07	0.93	0.87	0.78	0.69	0.66
51–56	1.16	0.88	1.24	0.90	1.00	0.75	0.77	0.63
57–63	0.70	0.49	0.68	0.46	0.55	0.39	0.45	0.33
64–70	1.18	0.76	1.24	0.80	0.86	0.68	0.78	0.57
71–77	0.86	0.71	0.80	0.68	0.65	0.59	0.52	0.50

**DAFLA**		***k*_HT_**	***k*_40_**	***k*_40_**	***k*_60_**	***k*_60_**	***k*_80_**	***k*_80_**

29–35		0.96	1.17	1.07	0.96	0.93	0.80	0.81
36–43		0.99	1.10	1.06	0.88	0.91	0.69	0.78
44–50		0.89	1.05	1.02	0.85	0.86	0.68	0.73
51–56		0.76	1.07	1.02	0.86	0.85	0.66	0.72
57–63		0.70	0.97	0.94	0.79	0.80	0.64	0.67
64–70		0.64	1.05	1.05	0.73	0.89	0.66	0.75
71–77		0.83	0.93	0.96	0.76	0.83	0.60	0.70


*DAFLA indicates day after the first true leaf appearance*.

### Architectural Effects of Salinity

The reduction (%) in total leaf area under salinity was similar between LT and HT conditions (data not shown). Both under LT and HT conditions, the architectural effects on reducing dry mass production (*R*_a,x_ in Eq. 4a) decreased with time after exposure to salinity (**Table 3**). Architectural effects depended on temperature regimes and increased with salinity level. In general, they were stronger at HT than at LT. For example, architectural effects at 80 mM NaCl over the whole growing period reduced dry mass production by 9.7 and 21.9% under LT and HT, respectively. Furthermore, architectural effects did not change strongly with salinity level under LT conditions while under HT they were twice as high as at 80 than at 40 mM NaCl (**Table 3**).

**Table 3:** Architectural (*R*_a,x_, Eq. 4a) and non-architectural effects (*R*_n,x_, Eq. 4b) on reducing dry mass production under *x* mM NaCl at 22/18°C (LT) and 32/28°C (HT) day/night temperature conditions.

	*R*_a,40_ (%)		*R*_a,60_ (%)		*R*_a,80_ (%)		*R*_n,40_ (%)		*R*_n,60_ (%)		*R*_n,80_ (%)	
						
DAFLA	LT	HT	LT	HT	LT	HT	LT	HT	LT	HT	LT	HT
29–35	23.2	21.2	24.9	27.8	27.5	34.4	-11.1	-4.9	2.6	4.4	12.8	11.0
36–43	11.7	13.7	16.4	18.3	15.6	24.0	-8.5	-5.2	9.8	7.3	25.7	16.6
44–50	12.1	8.9	12.5	13.3	12.7	18.7	-4.6	-2.0	13.0	11.8	27.9	21.7
51–56	13.2	9.0	12.3	12.7	12.4	17.8	-5.9	-1.7	12.4	12.9	29.5	22.8
57–63	4.3	1.1	4.2	6.5	5.5	12.8	1.5	5.3	19.9	18.7	33.8	28.0
64–70	11.3	13.0	9.4	17.4	8.4	22.8	-3.7	-3.1	24.0	10.1	31.3	20.0
71–77	0.9	5.7	0.4	13.2	0.8	20.3	4.2	-2.1	25.6	13.7	37.6	22.5
29–77	8.8	10.4	8.9	15.8	9.7	21.9	-2.7	1.2	16.8	10.5	29.0	19.0


*DAFLA indicates day after the first true leaf appearance*.

### Non-architectural Effects of Salinity

Non-architectural effects increased with salinity level. In contrast to architectural effects, non-architectural effects (*R*_n,x_ in Eq. 4b) increased with time and were higher at LT than at HT for the whole growing period. Both architectural and non-architectural effects increased with salinity level. They were close to zero under 40 mM NaCl and increased to 29 and 19% under 80 mM NaCl at LT and HT, respectively. Furthermore, the sum of architectural and non-architectural effects was similar to the measured reduction of shoot dry mass under salinity (see High Temperatures Aggravate Salinity Effects on Shoot Dry Mass in the results).

### Analyses of Architectural Traits

Shoot dry mass was most sensitive to leaf number and the reduction of leaf number decreased total leaf area almost linearly (**Table 4**). The sensitivity of dry mass production to architectural traits was temperature dependent. For example, while the change in leaf elongation rate (*c*_El,max_ in Eq. S2) influenced the total leaf area in the same magnitude at both LT and HT conditions, shoot dry mass at LT was not affected but reduced by up to 20% at HT (**Table 4**). Shoot dry mass was less sensitive to internode length and leaf angle at HT than at LT. Changes in leaf angle had less influence on dry mass production under 80 mM NaCl than under 40 mM NaCl. The reduction of dry mass production was linearly related to the light interception by the canopy (Supplementary Figure S1). The change in light interception explained 85 and 76% of the reduction in dry mass production at LT and HT, respectively. Furthermore, both at LT and HT conditions, the sensitivity of shoot dry mass to internode length was similar at 0, 40, and 80 mM NaCl (Supplementary Table S3).

**Table 4:** Relative shoot dry mass (*W*_sh,x_, % of the reference canopy architecture) and total leaf area (*A*_s_, % of the reference canopy architecture), and light transmittance through the canopy (*Q*_T_/*Q*_0_).

Traits	Salinity (mM NaCl)	Magnitude of change of trait		LT		HT		
				
			*W*_sh,x_ (%)	*A*_s_ (%)	*Q*_T_/*Q*_0_ (%)	*W*_sh,x_ (%)	*A*_s_ (%)	*Q*_t_/*Q*_0_ (%)
LN	40	88%	93.1	87.7	47.8	91.1	87.5	53.1
		76%	84.5	76.3	51.4	80.7	75.8	56.8
		64%	75.2	64.3	55.2	69.6	63.4	62.0
	80	76%	82.3	76.3	55.0	79.1	75.9	67.4
		52%	61.4	53.1	64.2	73.1	50.1	73.1
		28%	27.1	25.2	84.6	12.3	18.1	99.5
*c*_ El,max_	40	-0.0003	99.6	111.2	43.6	104.7	112.2	46.4
		-0.00045	99.8	105.5	44.3	102.6	106.0	47.5
		-0.00075	100.0	94.6	46.3	97.1	94.2	51.6
		-0.0009	99.9	89.5	47.0	93.9	88.6	54.2
	80	-0.0003	101.5	125.7	45.0	115.2	128.3	50.0
		-0.00045	101.1	112.4	47.0	107.9	113.6	54.2
		-0.00075	97.1	88.4	50.0	90.8	87.4	64.6
		-0.0009	92.1	77.7	54.3	80.9	75.7	71.1
IL	40	92%	96.6	100	45.6	98.3	100	49.4
		84%	93.0	100	45.7	96.0	100	49.3
		76%	89.1	100	46.1	93.7	100	49.1
	80	84%	93.8	100	48.8	96.4	100	58.5
		68%	85.7	100	49.1	91.8	100	58.0
		52%	76.2	100	50.5	85.6	100	57.6
𝜃	40	70%	123.7	100	29.7	106.0	100	46.6
		85%	114.3	100	33.7	104.0	100	45.9
		115%	86.4	100	56.7	91.3	100	58.1
		130%	76.8	100	66.4	79.3	100	70.4
	80	70%	114.4	100	39.2	102.4	100	58.7
		85%	109.7	100	40.1	102.4	100	57.2
		115%	86.9	100	60.8	94.6	100	64.4
		130%	75.8	100	70.7	86.1	100	72.8


*Values are simulated data on day 77 after the appearance of the first leaf to different architectural traits under 22/18°C (LT) and 32/28°C (HT) day/night temperature. In all cases, standard errors were smaller than 3%. The magnitudes of change in architectural traits are similar to the reported magnitude reported in the literature (Supplementary Table S1)*.

*LN, leaf number, in percentage of leaf number under 0 mM NaCl; *c*_*El,max*_, parameter for salinity effect on leaf area in Eq. 2 (reference value = -0.0006); IL, internode length, inpercentage of IL under 0 mM NaCl; 𝜃, leaf angle, in percentage of 𝜃 under stress conditions; *W*_*sh,x*_, shoot dry weight in percentage of the *W*_*sh,x*_ under the correspondent stress conditions; *A*_*s*_, total leaf area in percentage of the *A*_*s*_ under non-stress conditions; *Q*_*t*_/*Q*_0_, light transmittance through the canopy in percentage of the light intensity above the canopy*.

### DISCUSSION

### Stress Combinations have Opposite Effects on Different Traits

The results from our model analyses support the hypothesis that (1) architectural effects of salinity are more prominent under HT than under LT, especially under high salinity and (2) non-architectural effects are more prominent under LT than under HT (**Table 3**).

The primary architectural effects of salinity are the reduction of leaf area and leaf angle (Jones and El-Beltagy, 1989; Li and Stanghellini, 2001; Najla et al., 2009). The reduction in shoot dry mass under low salinity (40 mM NaCl, 6.1 and 19.9% for LT and HT conditions, respectively) may be mostly explained by the architectural effects (8.8 and 10.4% for LT and HT conditions, respectively). It is interesting to note that the architectural effects decreased with time (**Table 3**). This indicates that architectural effects of salinity are stronger in a younger open canopy and decrease with canopy closure, similar to the impacts of architectural traits on dry mass production under non-stressed conditions (Chen et al., 2014). The fact that canopies under LT had higher leaf areas (**Figures 3A,B**) and were more closed also explains why architectural effects of salinity are smaller under LT than under HL. Interestingly, under LT condition, the increase of salinity level from 40 to 80 mM NaCl reduced the total leaf area by additional 20% (**Figures 3A and 4A**) but this reduction in leaf area only resulted in an extra 0.9% of architectural effects on dry mass production (**Table 3**). This could be explained by the fact that light interception of the canopies under 40 and 80 mM NaCl were about equal (55 and 52%, respectively, **Table 4**), indicating that the architectural effects of salinity at LT were mainly an effect of leaf angle, but not of leaf area. This is also the reason why shoot dry weight was less sensitive to the leaf elongation (parameter *c*_El,max_ in Eq. 2) under LT (**Table 4**).

The primary non-architectural effects of salinity are the reduction of stomatal conductance, mesophyll conductance and photosynthetic capacity due to ion toxicity (Delfine et al., 1999; Munns and Tester, 2008; Pérez-López et al., 2012). These non-architectural effects of salinity can be reduced by increasing temperature. For example, stomatal and mesophyll conductance increase with leaf temperature (Carmo-Silva and Salvucci, 2012; Evans and von Caemmerer, 2013) and Na^+^ uptake rate of tomato plant is reduced under HT (Rivero et al., 2014). Therefore, our results are in accordance with recent findings of Rivero et al. (2014). Several studies found negative effects of HT (>35°C) on mesophyll conductance and biochemical capacity that reduce leaf photosynthesis (Yamori et al., 2010; Egea et al., 2011). However, day temperature higher than 35°C only rarely occurred during our experiment. Our results suggest that positive effects of HT on plant responses to salinity (lower sodium accumulation and higher photosynthesis) could not counterbalance the negative effects of HT on canopy architecture and light interception under salinity. Therefore, HT, in total, aggravates the salinity effects on dry mass production. Our results reconcile the findings of Keleks and Oncel (2002) and Rivero et al. (2014) and highlight (1) the differences in temperature effects on canopy architecture and light use efficiency under salinity and (2) the importance of plant architecture in studying the plant responses to the environments.

### Methodological Considerations for Dissecting the Architectural and Non-architectural Effects

The measured data showed strong allometric relationships between shoot dry weight and total leaf area (**Figures 4A,B**). The simulated results from a model, where the leaf growth dynamics, distribution of leaves in the space, light interception and photosynthesis are described precisely, should also be able to describe these allometric relationships. Since we have carefully evaluated our architectural model and shown that both at organ and canopy levels, our architectural model may predict the dynamic changes of plant architecture with very high accuracies (**Figure 2**; **Table 1** and Chen et al., 2014), we may estimate the relative canopy light use efficiency (*k*_T,x_ in Eq. 3a) based on the measured allometric relationships between shoot dry mass and total leaf area. To assure that these estimations are plausible, we carefully examined the prerequisites and the results of this new method.

Very importantly, we want to emphasize the prerequisite of this method. Model analyses have shown that dry mass production can be strongly influenced by architectural traits while the simulated leaf area maintains the same (Chen et al., 2014). This indicates that the three-dimensional distribution of the leaves in the space may influence the allometric relationship between shoot dry mass and total leaf area. Therefore, not only the accurate predictions for total leaf area, but also the accurate distributions of the leaves in the space are crucial for the simulated results reflecting the measured allometry. Hence, the mismatch between simulated and measured allometric relationships between shoot dry mass and total leaf area may be the results of both an inaccurate distribution of leaves and an inaccurate model for photosynthesis. Therefore, the method proposed in this paper may only be applicable for the dynamic FSPM, where the details in three-dimensional distribution of leaves can be simulated precisely (Evers et al., 2010; Cieslak et al., 2011; Kahlen and Stützel, 2011). This is also the reason why architectural traits in our model should be evaluated by the measured data before estimating *k*_T,x_. Therefore, this method may not be applied to traditional crop models, where the architectural information of the plant is missing. Further concern would be that the allometric relationship between total leaf area and shoot dry mass can be influenced by the dry mass allocation between shoots and roots (Poorter et al., 2012). Form our experiments, shoot dry mass consisted of 84–91% of the plant dry mass (shoot + root, data not shown). However, the differences between treatments and developmental stages were in the most cases insignificant. To avoid over parameterization, the average (87% of plant dry mass are partitioned into shoot) was taken in the simulation. This might result in a slight error in our model analyses (probably up to 0.05 of *k*_T,x_ by our educational guess).

The relative canopy light use efficiency, *k*_T,x_, and effects of HT and salinity on it, *k*_HT_ and *k*_x_, respectively, (Eqs 3a,b, **Table 2**) showed several trends: (1) *k*_HT_ was smaller than one; (2) *k*_LT,0_ and *k*_HT,0_ decreased with time; (3) *k*_x_ decreased with increasing salinity level; (4) *k*_x_ decreased with time after exposure to salinity; (5) the magnitude of the decrease in *k*_x_ with time under 60 and 80 mM NaCl was stronger than it under 40 mM NaCl; and (6) *k*_x_ under LT, especially under 80 mM NaCl, was smaller than it under HT. Because that all this trends can be well-explained by the findings reported in the literature, we consider that our estimations of the relative canopy light use efficiency and the following quantification of architectural and non-architectural effects were plausible. First, under control conditions, tomato has its best photosynthetic performance at around 25°C (Gent and Seginer, 2012; Qian et al., 2012). The average day temperatures in the greenhouses were 23 and 29°C for LT and HT conditions, respectively. The fact that the average day temperature in LT was closer to the optimal temperature than it was in HT may explain that *k*_HT_ was smaller than one throughout the whole growing period. Secondly, the function for canopy light use efficiency [𝜀(*I*_abs_(*t*)) in Eq. 1] was parameterized from a mature tomato canopy (Warren-Wilson et al., 1992). Before day 43, tomato plants were in the young developmental stage with less than 23 leaves and with plant height shorter than 120 cm. This explains that *k*_T,0_ was larger than one before day 43 because young canopy, which has a higher photosynthesis capacity (Qian et al., 2012). Therefore, canopy light use efficiency decreases with the age of the canopy. This also explains that *k*_HT,0_ and *k*_HT,0_ with time. Both osmotic and ionic components of salinity reduce the photosynthesis capacity of the plants and the magnitude of this reductions increase with the salinity level in the nutrient solution (James et al., 2002; Maggio et al., 2007; Munns and Tester, 2008; Rajendran et al., 2009; Pérez-López et al., 2012). This explains the reduction of *k*_x_ with increasing salinity level and with time after exposure to salinity (**Table 2**). Furthermore, the magnitude of the decrease in *k*_x_ with time under 80 mM NaCl was stronger than it under 40 mM NaCl. This indicates that the ionic effect appeared faster and was more prominent under higher salinity. Finally, that *k*_x_ under LT was smaller than it under HT can be explained by the recent finding that tomato grown under LT accumulates more Na^+^ in leaves than grown under HT (Rivero et al., 2014). This indicates that leaves grown under HT may maintain low Na^+^ concentrations and, therefore, maintain their light use efficiency.

### Contributions of Architectural Traits on Yield Reduction

The decrease in leaf appearance rate and leaf number under salinity had the strongest effects on reducing total leaf area and dry mass production (**Table 4**). This indicates that maintaining young leaf production under salinity stress is a key architectural trait for salinity tolerance. Furthermore, maintaining young leaf production may also counterbalance the leaf senescence due to the ionic effect (Munns and Tester, 2008). The changes in leaf angle and internode length may also result in up to 25% differences in dry mass production (**Table 4**). This would partly explain the negative relationship between salt tolerance and salt-induced increases in leaf angle (Jones and El-Beltagy, 1989). Changes in leaf angle affected the light interception by the canopy by up to 35% while changes in internode length, in contrast, affected the light interception by the canopy by only 8% (**Table 4**). The light interception by the canopy may explain 85% of the effects of leaf angle on shoot dry mass while no relationship was found between the effects of internode on light interception and on shoot dry weight (Supplementary Figure S2). Reduction in internode length decreases the distance between leaves and enhances the self-shading (Takenaka, 1994; Sarlikioti et al., 2011), which resulted in a less efficient light use in the canopy (Chen et al., 2014).

## Conclusion

High temperatures aggravate the negative effects of salinity on dry mass production via plant architecture and light interception but ameliorate the salinity effects on canopy light use efficiency. These results highlight the different temperature effects on physiological and morphological responses to salinity and the importance of plant architecture in studying plant responses to environmental changes at canopy level. Our analyses suggest that leaf angles influence light interception more than light distribution, and that changes in internode length have stronger effects on light distribution than on light interception. Furthermore, our model analyses enable us to dissect the architectural effect from non-architectural effect of salinity, which is impossible to be done experimentally because in reality both effects occur together under osmotic stress.

## Conflict of Interest Statement

The authors declare that the research was conducted in the absence of any commercial or financial relationships that could be construed as a potential conflict of interest.
