# Supplementary material for: High temperature and vapor pressure deficit aggravate architectural effects but ameliorate non-architectural effects of salinity on dry mass production of tomato
Source: Front Plant Sci. 2015 Oct 20;6:887. doi: 10.3389/fpls.2015.00887 (PMC4612157; doi:10.3389/fpls.2015.00887)

***Supplementary Material***

**High temperature and vapor pressure deficit aggravate architectural effects but ameliorate non-architectural effects of salinity on dry mass production of tomato**

**Tsu-Wei Chen^1^*, Thi My Nguyet Nguyen^1^, Katrin Kahlen^2^, Hartmut Stützel^1^**

^1^ Leibniz Universität Hannover, Institute of Horticultural Production Systems, Hannover, Germany

^2^ Geisenheim University, Department of Vegetable Crops, Geisenheim, Germany

*** Correspondence:**

Tsu-Wei Chen, Leibniz

Universität Hannove

Institute of Horticultural Production Systems

Herrenhäuser Straße 2

30419 Hannover, Germany.

[chen@gem.uni-hannover.de](mailto:chen@gem.uni-hannover.de)

**Supplementary Information**

*Model description*

To simulate the plant architecture under salinity stress, salinity effects on tomato architectural traits were implemented into the dynamic functional-structural plant model of tomato proposed by Chen *et*  *al.* (2014). Leaf elongation rate *E*_l_(*t*, *TS*, *r*) (cm d^-1^) at leaf rank *r*, at a given leaf temperature sum, *TS* (°Cd, calculated by accumulating the difference between the average air temperature and the base temperature each day from the date of leaf appearance), and at time *t* was calculated as the product of the maximum leaf elongation rate of the leaf at rank 8, *E*_l,max_(*t*) (cm d^-1^), the normalized effect of temperature sum, *E*_l,norm_(*TS*), and a normalized rank effect, *R_l_*_,norm_(*r*) (Chen *et al.* 2014):

*E*_l_(*t*, *TS*, *r*) =*E*_l,max_(*t*)**^.^***E*_l,norm_(*TS*)**^.^***R*_l,norm_(*r*) (S1)

*E*_l,max_(*t*) was a function of temperature (*T*(*t*), °C), vapor pressure deficit (VPD(*t*), kPa) and the salt concentration *S*_S_ (mM NaCl) in the nutrient solution (Reymond *et al.*, 2003):

*E*_l_*_,_*_max_(*t*) = (*T*(*t*)-*T*_b_)(*a*_El,max_+*b*_El,max_·VPD(*t*)+*c*_El,max_·*S*_S_) for *T*_b_≤ *T*(*t*) ≤*T*_opt_ (S2a)

*E*_l_*_,_*_max_(*t*) = (2*T*_opt_-*T*(*t*)-*T*_b_)(*a*_El,max_+*b*_El.max_**^.^**VPD(*t*)+*c*_El,max_·*S*_S_) for *T*(*t*) >*T*_opt_ (S2b)

where *T*_b_  and *T*_opt_ are base and optimal temperatures, respectively. When *T*(*t*) is above *T*_opt_, *E*_l,max_(*t*) decreases at the same rate as it increases in the range of temperatures below the *T*_opt_. Normalized temperature effect, *E*_l,norm_(*TS*), normalized rank effects, *R*_r_*_,_*_norm_(*r*), and parameters *T*_b_ (6.8°C), *T*_opt_ (28 °C), *a*_El,max_ (0.318) and *b*_El.max_ (-0.029) were as described in Chen *et al*. (2014). The new parameter *c*_El,max_ (-0.0006±0.00003), describing the salinity effects on leaf elongation, was estimated from the data of Expt. 3, where the light intensity in the growth chamber was similar to typical greenhouse production conditions in the spring in Germany. The normalized effect of temperature sum, *E*_l,norm_(*TS*) in Eqn. S1, was considered as a bell shaped function depending on leaf temperature sum:

*E*_l,norm_(*TS*) = exp(-0.5((*TS*-*TS*_l,max_)/*h*_l_)^2^) (S3)

where *TS*_l,max_ (°Cd) is the temperature sum required by a leaf to reach its maximum elongation rate. Normalized rank effects, *R*_l_*_,_*_norm_(*r*) in Eqn. S1, on leaf elongation were assumed to follow a bell shaped function for ranks below 14:

*R*_l_*_,_*_norm_(*r*) = exp(-0.5((*r* -*R*_max_)/*h*_r_)^2^) (S4)

where *R*_max_ is the rank where a leaf has the maximum leaf length. Due to the short duration of the growth chamber experiments the measurement of rank effects could be done only for leaves on ranks 1 to 13. The maximum elongation rate *E*_l,max_ of leaves above rank 13 was assumed to be the same as of the leaves at rank 13.

Leaf length *L*_l_(*t*) at time *t* (cm) was calculated as the cumulative *E*_l_. The area of a leaf, *A*_l_(*t*) (cm^2^), was computed based on the allometric relationship between leaf length and area for this specific cultivar:

*A*_l_(*t*) = *a*_Al_ · *L*_l_(*t*) ^g^ (S5)

where *a*_Al_ and *g* are empirical coefficients. Salinity, temperature and their combination have no significant effect on these coefficients, similar to the results in cucumber (see Expt. 2 in Kahlen & Stützel, 2007). Leaf appearance rate at time *t*, *R*_l_(*t*) (leaf d^-1^) was:

*R*_l_(*t*) = *a*_r_·ln(*T*(*t*)) – *b*_r_ for *T*(*t*) ≤ 30°C (S6a)

*R*_l_(*t*) = *R_l_*_max_ for *T*(*t*) > 30°C (S6b)

where *a*_rl_ and *b*_rl_ are empirical parameters and if *R*_l_(*t*) reached its maximum value, *R*_lmax_, at 30°C, a further increase in temperature did not increase *R*_l_(*t*). The number of leaves was calculated as the integral of *R*_l_ over time. Leaf angle, *θ* (°), and leaf curvature, *C_l_* (°) were assumed to be leaf length dependent and followed logistic and linear functions for leaf angle and curvature, respectively.

*θ* = *a*_θ_**^.^**(1-exp(-(*b*_θ_**^.^** *L*_l_))) (S7)

*C*_l_ = *a*_Cl_-*b*_Cl_**^.^***L*_l_ for *L*_l_ ≤ 50 cm (S8b)

*C*_l_ = *a*_1Cl_+*b*_1Cl_·*L*_l_ for *L*_l_ > 50 cm (S8b)

where *a*_θ_, *b*_θ_, *a*_Cl_ and *b*_Cl_,are empirical coefficients. Internode elongation rate, *E*_i_(*t*) at time *t* (cm d^-1^), was modeled as the product of maximum internode elongation rate, *E*_i,max_(*t*) (cm d^-1^) and normalized internode elongation rate, *E*_i,norm_. *E*_i,max_(*t*) was computed similarly to *E*_l,max_(*t*) but was considered to be dependent on temperature and photosynthetically active radiation (PAR) above the canopy (Kahlen & Stützel, 2011):

*E*_i,max_(*t*) = (*T*(*t*)-*T*_bi_)(*a*_Ei,max_-*b*_Ei,max_**^.^**PAR(*t*)) (S9)

*E*_i,norm_(*TS*) = exp(-0.5((*TS*-*TS*_i,max,_)/*h*_i_)^2^) (S10)

where *T*_bi_ is the base temperature for internode growth. It was derived using the same procedure as base temperature for leaf growth. *TS* is temperature sum (°Cd) and *TS*_i,max_ is the temperature sum when the internode reaches its maximum elongation rate (°Cd). PAR(*t*) is photosynthetically active radiation of day *t* (µmol m^-2^ s^-1^). The parameters *h*_i_, *a*_Ei,max_ and *b*_Ei,max_ are shape coefficient. Internode length at time *t*, *L*_i_(*t*) (cm), is the accumulation of *E*_i_(*t*).

The effects of salinity on other architectural parameters, *X*, including leaf appearance rate (*R*_l_ in Eqn. S6), leaf angle (*θ* in Eqn. S7), leaf curvature (*C*_l_ in Eqn. S8) and maximum internode elongation rate (*E*_i,max_ in Eqn. S9), were quantified by:

*X* = (1+ *α*_x_*S*_S_)∙*X*_0_ (S11)

where *S*_S_ is the salinity level in the root zone (mM NaCl), *α*_x_ is an experimentally derived parameter describing the change of *X* due to salinity and *X*_0_ is the value of each architectural parameter at non-stress conditions. All results from Expts. 1-3 showed that salinity stress had no influence on leaf appearance rate, maximum internode elongation rate and leaf curvature of tomato cultivar Pannovy and there was no interaction between environmental factors (temperature, light, and VPD) and salinity. Therefore, parameters *α*_x_ in Eqn. S11 were zero for these traits. Leaf angles of salt-treated plants were on average 15.3% higher than in the control, with no significant difference between salinity levels. Therefore, the term (1+ *α*_x_*S*_S_) in Eqn. S11 for leaf angle was replaced by 1.153 for simulations under all salt stress conditions.

## Supplementary Tables

**Table S1**. Reported magnitudes of morphological changes in tomato under salinity stress

|  | Morphological change (per 10 mM NaCl in solution) | | | |  |
| --- | --- | --- | --- | --- | --- |
| Morphological traits | Leaf number | Leaf area | Stem length | Leaf angle | Reference |
| *Cultivar* |  |  |  |  |  |
| Marmara | ±0% | -7.4% | -4.0% | - | (Najla *et al.*, 2009) |
| Licata F1-COIS | -9.0% | -10.9% | - | - | (Maggio *et al.*, 2007) |
| Cois HC01 | -5.6% | -7.5% | - | - | (Maggio *et al.*, 2004) |
| Rio Grande | - | -3.0% | -1.8% | - | (Zribi *et al*., 2009) |
| Daniela | -1.7% | -3.7% | -3.3% | - | (Romero-Aranda *et al*., 2001) |
| Moneymaker | -1.3% | -4.8% | -1.6% | - | (Romero-Aranda *et al*., 2001) |
| Chaser | +3.7% | -9.5-17.1% | - | - | (Li and Stanghellini, 2001) |
| Patio | -2.5% | - | -3.8% | +9.2° | (Shibli *et al.*, 2007) |
| Roma | -2.3% | - | -3.4% | +12.4° | (Shibli *et al.*, 2007) |
| Various genotypes | - | - | - | 0.7-2.4° | (Jones and El-Beltagy, 1989) |

**Table S2.** Summary of experimental conditions.

| Expt | Location |  | Set conditions | | | |
| --- | --- | --- | --- | --- | --- | --- |
|  |  | Salinity | Day/night | VPD | PAR | CO_2_ |
|  |  | (mM NaCl) | temperature (°C) | (kPa) | (µmol m^-2^ s^-1^) | (ppm) |
| 1 ^A^ | Growth chamber | 0, 20, 40, 60 | 17/13, 22/18,  26/22 & 30/26 | 0.8 | 300 | 380 |
| 2  ^B^ | Growth chamber | 0, 40, 60, 80 | 22/18 | 0.4, 0.8 & 1.2 | 300 | 380 |
| 3 ^C^ | Growth chamber | 0, 40, 60, 80 | 22/18 | 0.8 | 300, 500 & 700 | 380 |
| 4 ^D^ | Greenhouse | 0, 40, 60, 80 | 22/18 | 1.1 | - | ambient |
| 5 ^E^ | Greenhouse | 0, 40, 60, 80 | 22/18  32/28 | 1.39^F^  2.01^F^ | -  - | ambient  ambient |

^A^:sowing on 5 Aug. 2008, transplanting to hydroponic system on 22 Aug. 2008. ^B^: sowing on 23 Sep. 2008, transplanting to hydroponic system on 8 Oct. 2008. ^C^: sowing on 23 Feb. 2009, transplanting to hydroponic system on 28 Mar. 2009. ^D^: sowing on 11 May 2009; seedlings were transplant to the greenhouse on 29 May 2009 and salt was applied in the solution on 2 Jun. 2009.^E^: sowing on 22 Mar. 2010; seedlings were transplant to the greenhouse on 14 Apr. and salt was applied in the solution on 18 Apr. 2010, 12 DAFLA.^F^: average values in the greenhouses.

**Table S3.** Sensitivity of shoot dry mass to internode length on day 77 after the first leaf appearance under 22/18°C (LT) and 32/28°C (HT) day/night temperature conditions. Numbers are means with standard error in parentheses.

|  |  | Shoot dry mass (% of reference) | | |
| --- | --- | --- | --- | --- |
| Conditions | Relative internode length | 0mM NaCl | 40 mM NaCl | 80 mM NaCl |
| LT | 0.7 | 88.1 (2.0) | 85.8 (1.4) | 86.7 (0.9) |
|  | 1.0 | 100.0 (2.5) | 100.0 (1.4) | 100.0 (1.0) |
|  | 1.3 | 109.2 (2.5) | 110.5 (1.5) | 107.3 (0.8) |
| HT | 0.7 | 92.4 (1.2) | 91.6 (0.7) | 92.4 (0.4) |
|  | 1.0 | 100.0 (1.2) | 100.0 (0.6) | 100.0 (0.5) |
|  | 1.3 | 104.9 (1.1) | 105.3 (0.6) | 104.3 (0.3) |

##
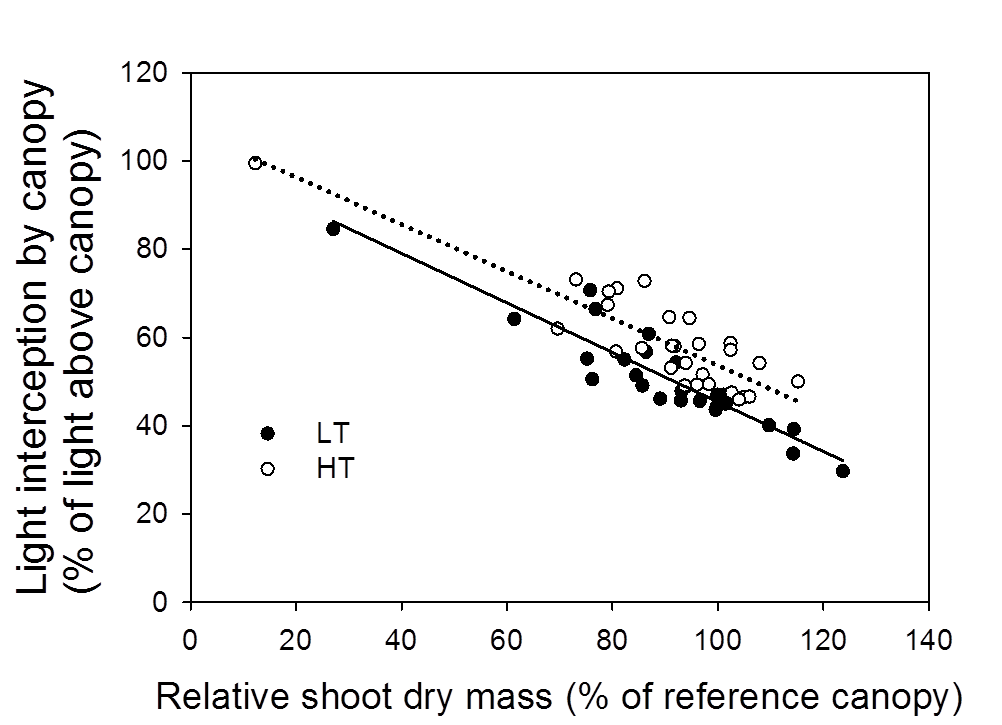
Supplementary Figures

**Figure S1.** Relationship between relative shoot dry mass and light interception at 22/18°C (LT, closed symbols and solid line, y = -0.56x + 101.52, *R*² = 0.85) and 32/28°C (HT, open symbols and dotted line, y = -0.53x + 106.97, *R*² = 0.76) day/night temperature regimes (derived from Table 5).


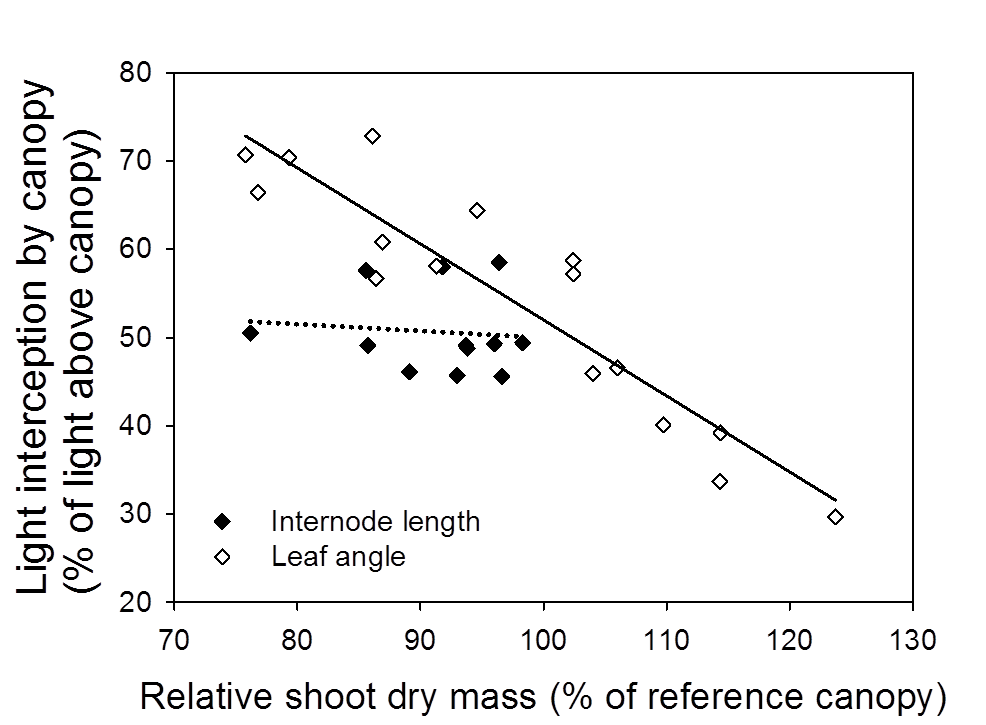


**Figure S2.** Relationship between relative shoot dry mass and light interception for internode length (closed symbols and dotted line, y = -0.077x + 57.66, *R*² = 0.01) and leaf angle (open symbols and solid line, y = -0.86x + 138.1, *R*² = 0.85) day/night temperature regimes (derived from Table 5).


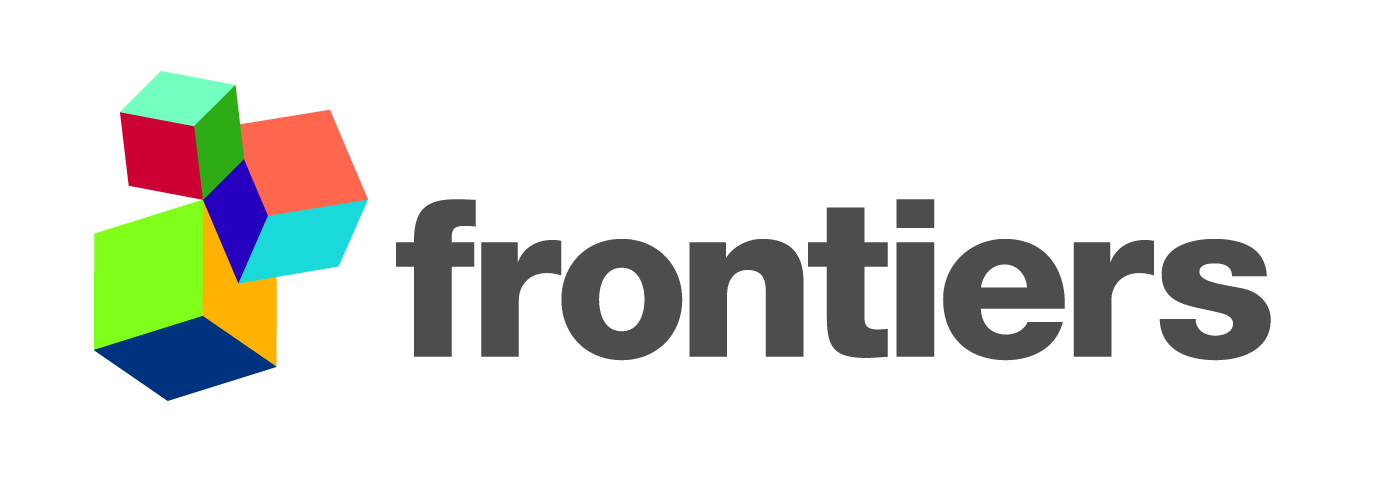

Supplement: Supplementary file 1 [file Data_Sheet_1.DOCX]
